# Unusual Presentation of Postprandial Sinus Tachycardia in Pregnancy

**DOI:** 10.7759/cureus.26425

**Published:** 2022-06-29

**Authors:** Sarah Stavros, Ana M Pinon, Kevin Gates, Peter Khamvongsa

**Affiliations:** 1 Obstetrics and Gynecology, Florida International University (FIU) Herbert Wertheim College of Medicine, Miami, USA

**Keywords:** third trimester complications, pregnancy related tachycardia, problems during pregnancy, pregnancy trimester third, swallowing-induced atrial tachycardia, prist, pregnancy related inappropriate sinus tachycardia

## Abstract

This report describes the clinical presentation and systematic workup of a 25-year-old primigravid woman presenting at 34 weeks gestation with symptoms of postprandial sinus tachycardia. Differentials included pregnancy-related inappropriate sinus tachycardia (PRIST) and swallowing-induced atrial tachycardia. PRIST has favorable outcomes for both the mother and baby, although the symptoms can be distressing and lead to the overutilization of healthcare resources. The literature describes symptom onset as sporadic with no definitive triggers, often occurring in the later trimesters of pregnancy. Swallowing-induced atrial tachycardia, which fits with postprandial symptoms seen in this patient, is a distinct entity most commonly diagnosed in middle-aged men. Management included psychosocial support and pharmacologic treatment with labetalol. The patient’s course was benign with an uncomplicated delivery at 38 weeks, complete resolution of symptoms in the postpartum period, and successful discontinuation of pharmacologic management with no relapses. Current literature on postprandial sinus tachycardia in pregnant women is limited, and the best treatment method is unknown. This report extends current knowledge on pregnancy-related postprandial sinus tachycardia and provides a framework for advances in the management of this clinical presentation.

## Introduction

Expected changes in the cardiovascular system during pregnancy include elevated heart rate and increased cardiac output when compared to prepregnancy values [1]. Physiologic heart rate during pregnancy should not exceed 95 beats per minute (bpm), and resting heart rates exceeding 100 bpm in pregnant patients could indicate a pathologic process. 

As tachycardia is common in pregnancy, the differential diagnosis is broad. Pregnancy-related inappropriate sinus tachycardia (PRIST) is a diagnosis of exclusion defined as sinus rates greater than 100 bpm at rest or greater than 90 bpm on average over 24 hours during pregnancy. The diagnosis is closely related to Inappropriate Sinus Tachycardia (IST), which typically presents sporadically in young women. Proposed mechanisms of PRIST include a hormonally mediated increase in sinus node sensitivity and an exaggerated response to physiologic cardiovascular changes normally experienced in pregnancy [2]. To diagnose IST, the tachycardia must not be due to underlying causes, and the patient must present with symptoms secondary to tachycardia, such as feelings of lightheadedness or palpitations [3].

Swallowing- or deglutition- induced atrial tachycardia presents more commonly in hemodynamically-stable middle-aged men without orthostatic change or structural abnormalities in cardiac or gastrointestinal systems [4]. This presentation has not yet been reported in pregnancy. Proposed mechanisms include stimulation of an adrenergic reflex in the esophagus while swallowing and mechanical stimulation of the left atrium by the distended esophagus [4]. The literature recommends conservative treatment with dietary changes to avoid food triggers (i.e., alcohol, caffeine) and consuming smaller bites of food. Pharmacologic treatment with beta-blockers and calcium channel blockers is warranted if symptoms become too frequent or don’t respond to behavioral changes [4]. The objective of this report is to describe the case of a 25-year-old primigravid woman who presented with symptoms of inappropriate sinus tachycardia at 34 weeks gestation, with tachycardia occurring postprandially. The top differentials for this patient included pregnancy-related inappropriate sinus tachycardia (PRIST) and swallowing-induced atrial tachycardia.

## Case presentation

The patient was a 25-year-old primigravid Hispanic woman at 34 weeks gestation who presented to the Obstetric Emergency Department (OB ED) with recurrent episodes of “lightheadedness and elevated heart rate.” She was prompted to come for evaluation from work due to dizziness, lightheadedness, and tunnel vision. At work, she reported her blood pressure (BP) ranged from 150-160/90-100, and her heart rate ranged from 140-150 bpm. The patient reported a two-month history of episodic tachycardia and dizziness, with occasional headaches, occurring 10-15 minutes after meals. She experienced these episodes three to four times daily, and the episodes occurred both postprandially and sporadically. She denied fever, syncope, temperature intolerance, weight loss, chest pain, headaches, and vision changes with these episodes. At the onset of the symptomatic episodes, she reported performing vagal maneuvers and/or carotid massage to bring her pulse down. The patient found the episodes distressing since they had never happened before or during pregnancy, and she was concerned about the potential impact on her and her baby’s health. She reported good fetal activity and denied signs of preterm labor, including contractions and vaginal bleeding.

The patient’s past medical history was positive for migraines without aura, not intractable, which did not require medications for relief or prevention. Her only reported medications included prenatal vitamins. She had been to all routine prenatal OB appointments. First trimester labs reported a red blood cell count of 4.49, hemoglobin 13.8, and hematocrit 40.5 (Table 1). See Table 1 for other collected lab values. She had a history of resolved minor COVID-19 infection more than a year before the presentation and reports complete resolution of symptoms without complications. She denied personal and family history of cardiac problems. She denied the use of alcohol, tobacco products, or other recreational drugs. She exercised for one hour twice a week and drank one to two cups of coffee daily. 

**Table 1:** Patient Lab Values g/dL: grams per deciliter; mg: milligram; pmol/liter: micromol/liter; mg/dL: milligrams per deciliter

Lab Test	Patient	Reference Range	Units
Hemoglobin	13.8	12.8 - 16.9	g/dL
Hematocrit	40.5	34.4 - 50.8	%
Red Blood Cell Count	4.49	34.4 - 50.8	%
VMA 24-hour urine	3.4	2 - 7	mg
T4	19.1	12 - 30	pmol/L
Fibrinogen	591	200 - 400	mg/dL
HIV Antibody	Negative	-	-
Rapid Plasma Reagin	Negative	-	-
Hepatitis B surface antibody	Reactive	-	-
Hepatitis C antibody	Negative	-	-
Rubella IgM Antibody	Positive	-	-

A review of symptoms was positive for shortness of breath and palpitations. Physical exam in the OB ED reported HR of 126 BPM and BP of 130/69. Her vital signs normalized with supine positioning in left lateral decubitus. A cardiac exam revealed regular rate and rhythm without murmurs, rubs, or bruits. The remainder of her physical exam revealed no thyromegaly, lungs clear to auscultation bilaterally, abdomen soft and non-tender, no calf tenderness, no extremity edema, and palpable peripheral pulses. The pelvic exam was deferred. Electronic fetal monitoring reported Category 1 fetal heart tracings that were reactive with accelerations, moderate variability, and no decelerations. Tocometry reported no contractions.

The patient was admitted under the care of her obstetrician for observation and telemetry monitoring to capture the episodes of tachycardia and the aforementioned associated symptoms. She was subsequently evaluated by cardiology and endocrinology to further evaluate possible causes for tachycardia such as PRIST, deglutition-induced atrial tachycardia, hyperthyroidism, preeclampsia, pheochromocytoma, and peripartum cardiomyopathy. Labs reported T4 of 19.1, 24-hour urinary protein of 256 with vanillylmandelic acid (VMA) 3.4, and fibrinogen level of 591 (Table 1). All other lab values, including liver function tests, serum chemistry, urinary catecholamines, urine toxicology, and morning cortisol were negative or within normal limits. The 12-lead EKG at rest was interpreted as sinus rhythm. When performed postprandially during a symptomatic episode, the EKG was interpreted as sinus tachycardia. An echocardiogram revealed normal left ventricular function, trace mitral, tricuspid, and pulmonic regurgitation, and trivial pericardial effusion that was not hemodynamically significant, with an estimated ejection fraction of 60%-65%. 

Following complete work-up, the patient’s differential diagnosis was narrowed down to PRIST and swallowing-induced atrial tachycardia. Hyperthyroidism was ruled down due to patient history, normal thyroid exam, and a normal T4. Although the patient reported elevated blood pressure readings at home, she was normotensive in the OB ED. Preeclampsia was ruled down due to unremarkable urine protein findings, and pheochromocytoma was ruled down secondary to low urinary catecholamines. Lack of abnormalities on echo with Doppler made the diagnosis of peripartum cardiomyopathy unlikely. 

Per cardiology’s recommendation, the patient was started on 100 mg labetalol twice daily to manage symptoms. Notably, labetalol is used for medical management of both PRIST and swallowing-induced tachycardia [2,4]. Following administration of labetalol, the patient was able to stand up, ambulate, and use the bathroom postprandially without tachycardia or other associated symptoms. Throughout her three-day hospitalization, she remained stable and was instructed to continue the labetalol 100 mg twice daily and follow up with her obstetrician for her 37-week appointment.

At her 37-week visit, all vital signs were within normal limits, and she reported that she was doing well but had taken leave from work to reduce her activity level and physical stressors. While the labetalol was preventing her tachycardic episodes, she reported that she had an episode of lightheadedness, possibly due to hypotension, that forced her to sit down while at the grocery store. At this time, the labetalol dosing was reduced to 50 mg twice daily to prevent overmedication and hypotension. Following adjustment of the labetalol, the patient had noncontributory follow-up leading up to delivery, including normotensive blood pressure readings in the office. She had an uncomplicated delivery via Cesarean section at 38 weeks and five days due to breech presentation. Labetalol was discontinued after delivery. At follow-up, one week postpartum, she reported complete resolution of her postprandial tachycardia. She was in sinus rhythm and normotensive without the need for anti-hypertensives. At follow-up, eight weeks postpartum, the patient and her baby were doing well with no further symptoms of postprandial or inappropriate tachycardia in the patient.

## Discussion

While this presentation is similar to both PRIST and swallowing-induced tachycardia, the timing of postprandial symptoms in pregnancy is unusual and is not mentioned in existing literature describing these conditions [2,4,5]. Despite this difference, our patient’s case shared similarities to existing documentation of PRIST, which share consistent features, including no cardiac symptoms before pregnancy, a benign course, and resolution in the post-partum period [2,5]. One case in the literature describes a 32-year-old woman who developed PRIST at 36 weeks gestation, was induced at 37 weeks secondary to distressing symptoms, and experienced resolution of symptoms by eight weeks postpartum. In a retrospective, observational cohort analysis of 19 pregnant patients with confirmed diagnoses of inappropriate sinus tachycardia, cardiac symptoms resolved for 17 of the 19 patients within one week of delivery [5]. Of the remaining two, one had symptom resolution within four months. The other patient was restarted on beta-blockers due to a subsequent pregnancy with continued symptoms. Of the 19 women, 18 delivered at term, and there were no adverse fetal outcomes. There were no instances of an acute coronary syndrome, heart failure, or thromboembolic or hemorrhagic complications during pregnancy. Despite the benign course, this analysis documented significant usage of healthcare resources for this cohort of patients experiencing PRIST. The analysis reported that 42% of the cohort presented to the ED on more than one occasion secondary to symptoms of PRIST, and 32% required hospital admission. While the patient, in this case, did not present to the ED on multiple occasions nor require readmission, her initial admission required an extensive workup. Ultimately, a thorough workup is necessary to rule out conditions with significant morbidity for both mother and baby. Rates of induction of PRIST patients in the aforementioned study were also elevated when compared to the overall rate of induction for the hospital (58% and 25%, respectively) [5]. This may have been avoided if there was more awareness surrounding the condition since it is mostly benign and resolves after delivery.

In the evaluation of a pregnant patient with symptoms of tachycardia, either sporadically or postprandially, we recommend conducting a thorough evaluation to exclude other mechanisms and tachyarrhythmia. The use of a Holter or loop monitor with patient feedback can correlate symptoms with triggers. Laboratory workup should include electrolytes, magnesium, calcium, phosphate, thyroid-stimulating hormone (TSH), plasma metanephrines, renal function testing, and cardiac biomarkers. The case of this patient was similar to the documentation of both PRIST and swallowing-induced atrial tachycardia in the literature, as patients presented with normal cardiac anatomy, but it is still important to assess for possible structural anomalies via echocardiogram. [3,4] Chest radiograph and lung perfusion scan may also be indicated if the differential includes a possible pulmonary embolism. Regarding treatment, emphasis should be placed on patient reassurance and psychosocial support. If symptoms are distressing to the patient, pharmacological treatment with labetalol can be initiated and continued until the resolution of symptoms, likely in the postpartum period [5].

Strengths of this report include frequent face-to-face follow-ups with the patient at her OB check-ups after discharge from the antepartum unit. Frequent follow-up allowed for close monitoring of the patient and her baby, patient updates regarding vital signs at home and efficacy of the pharmaceutical intervention, and the ability to modify recommendations as changes arose. The patient was able to get a full cardiac work-up during her initial inpatient stay, definitively ruling out other underlying cardiac causes. Because there is so little documentation describing presentations similar to this patient’s, one potential disadvantage of this report is the anecdotal fallacy, as it could be dangerous to overinterpret a single case. 

## Conclusions

Although the reports of PRIST and swallowing-induced tachycardia described in the discussion have favorable outcomes, the literature is limited. Incomplete awareness of these differentials can lead to an increase in maternal distress due to the lack of a definitive explanation and the distressing symptoms associated with tachycardia. While a thorough workup is necessary to rule out more serious causes, there may also be unnecessary overuse of healthcare resources due to repeat hospital admissions and inductions, which may not be necessary given the benign clinical findings and excellent prognosis of women presenting with PRIST.

This report adds to the scarce body of literature describing symptoms of inappropriate sinus tachycardia in pregnancy, specifically postprandially, with the hopes of increasing general knowledge regarding the presentation, course, prognosis, and most favorable treatments to healthcare providers.

## References

[1] Soma-Pillay P, Nelson-Piercy C, Tolppanen H, Mebazaa A (2016). Physiological changes in pregnancy. Cardiovasc J Afr.

[2] Belham M, Patient C, Pickett J (2017). Inappropriate sinus tachycardia in pregnancy: a benign phenomena?. BMJ Case Rep.

[3] Olshansky B, Sullivan RM (2019). Inappropriate sinus tachycardia. Europace.

[4] Mathew R, Green MS, Nery PB (2018). Swallowing-induced atrial tachycardia. CMAJ.

[5] Sharp A, Patient C, Pickett J, Belham M (2021). Pregnancy-related inappropriate sinus tachycardia: A cohort analysis of maternal and fetal outcomes. Obstet Med.

